# Supplementary material for: Secretome of Mesenchymal Stem Cells from Consecutive Hypoxic Cultures Promotes Resolution of Lung Inflammation by Reprogramming Anti-Inflammatory Macrophages
Source: Int J Mol Sci. 2022 Apr 14;23(8):4333. doi: 10.3390/ijms23084333 (PMC9032661; doi:10.3390/ijms23084333)
Supplement: Supplementary file 1 [file ijms-23-04333-s001.zip › ijms-1649054-supplementary.pdf]

International Journal of *Molecular Sciences*

Article

Secretome of Mesenchymal Stem Cells from Consecutive Hypoxic Cultures Promotes Resolution of Lung Inflammation by Reprogramming Anti-inflammatory Macrophages

Zhihong Xu [1], Lulu Lin [1], Yuxuan Fan [1], Céline Huselstein [2], Natalia De Isla [2], Xiaohua He [1], Yun Chen [1] and Yinping Li [1,*]

[1] Department of Pathophysiology, Hubei Province Key Laboratory of Allergy and Immunology, Taikang Medical School (School of Basic Medical Sciences), Wuhan University, Wuhan 430071, China; xuzhihong@whu.edu.cn (Z.X.); linlulu@whu.edu.cn (L.L.); fanyuxuan@whu.edu.cn (Y.F.); hexiaohua@whu.edu.cn (X.H.); yunchen@whu.edu.cn (Y.C.)
[2] UMR 7365 CNRS, Medical School, University of Lorraine, 54505 Nancy, France; celine.huselstein@univ-lorraine.fr (C.H.); natalia.de-isla@univ-lorraine.fr (N.D.I.)
* Correspondence: liyinping@whu.edu.cn; Tel: +86-27-6875-8727, Fax: + 86-27-6875-9222

Citation: Xu, Z.; Lin, L.; Fan, Y.; Huselstein, C.; De Isla, N.; He, X.; Chen, Y.; Li, Y. Secretome of Mesenchymal Stem Cells from Consecutive Hypoxic Cultures Promotes Resolution of Lung Inflammation by Reprogramming Anti-Inflammatory Macrophages. *Int. J. Mol. Sci.* **2022**, *23*, 4333. https://doi.org/10.3390/ijms23084333

Academic Editor: Lorenza Lazzari

Received: 7 March 2022
Accepted: 11 April 2022
Published: 14 April 2022

Publisher's Note: MDPI stays neutral with regard to jurisdictional claims in published maps and institutional affiliations.

Copyright: © 2022 by the authors. Submitted for possible open access publication under the terms and conditions of the Creative Commons Attribution (CC BY) license (https://creativecommons.org/licenses/by/4.0/).

Supplementary Materials:

The Supplementary Information includes the following information:

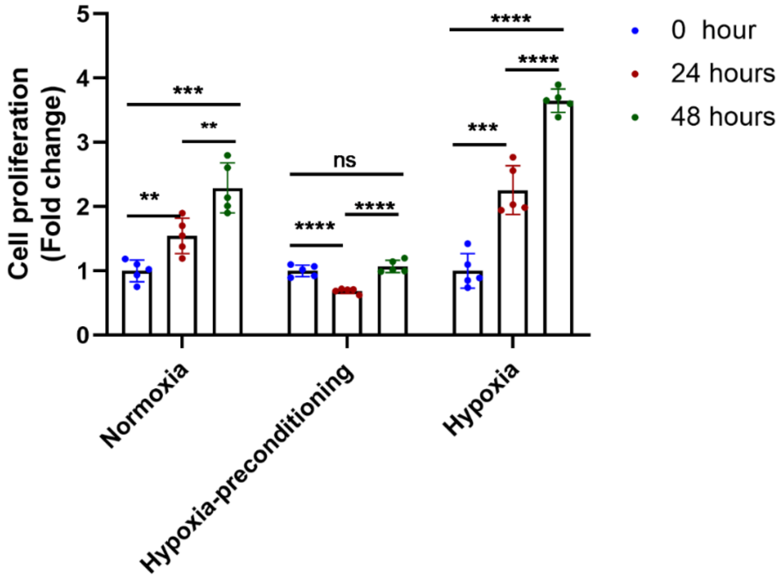

Figure S1. The proliferation rate of UC-MSCs under different modes of hypoxia treatment. Cells of passage 3 or passage 4 were used to analyze the proliferation rate of UC-MSCs at 0, 24, and 48 h through CCK8 assay. The OD value of cells at 0 h was set to 1-fold. ** $p < 0.01$, *** $p < 0.001$ and **** $p < 0.0001$. Normoxia, normoxic cultures; Hypoxia, consecutive hypoxic cultures; CCK8, cell counting kit-8; OD, optical density.

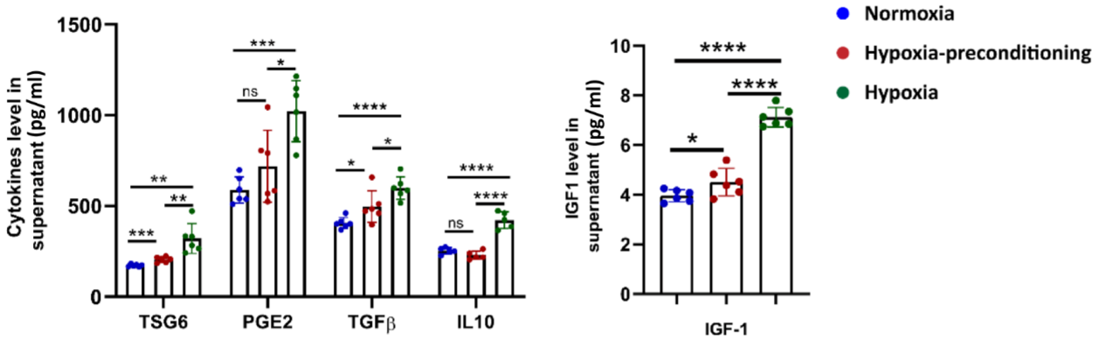

Figure S2. The levels of immunomodulatory factors in the conditioned medium from UC-. MSCs were analyzed by ELISA. Cells of passage 3-4 under different modes of hypoxia treatment were used to analyze factors (TSG6, PGE2, TGFβ, IL10, and IGF1) levels. * $p < 0.05$, ** $p < 0.01$, *** $p < 0.001$ and **** $p < 0.0001$. Normoxia, normoxic cultures; Hypoxia, consecutive hypoxic cultures.
